# Myc and Omomyc functionally associate with the Protein Arginine Methyltransferase 5 (PRMT5) in glioblastoma cells

**DOI:** 10.1038/srep15494

**Published:** 2015-11-13

**Authors:** Maria Patrizia Mongiardi, Mauro Savino, Laura Bartoli, Sara Beji, Simona Nanni, Fiorella Scagnoli, Maria Laura Falchetti, Annarita Favia, Antonella Farsetti, Andrea Levi, Sergio Nasi, Barbara Illi

**Affiliations:** 1 Institute of Cell Biology and Neurobiology, National Research Council (IBCN-CNR), Rome, Italy; 2 Nucleic Acids Laboratory, Institute of Molecular Biology and Pathology, National Research Council (IBPM-CNR) and Dept. of Biology and Biotechnologies, Sapienza University; 3 Istituto di Patologia Medica, Università Cattolica del Sacro Cuore, Rome, Italy; 4 Department of Experimental Oncology, Regina Elena National Cancer Institute, Rome, Italy

## Abstract

The c-Myc protein is dysregulated in many human cancers and its function has not been fully elucitated yet. The c-Myc inhibitor Omomyc displays potent anticancer properties in animal models. It perturbs the c-Myc protein network, impairs c-Myc binding to the E-boxes, retaining transrepressive properties and inducing histone deacetylation. Here we have employed Omomyc to further analyse c-Myc activity at the epigenetic level. We show that both Myc and Omomyc stimulate histone H4 symmetric dimethylation of arginine (R) 3 (H4R3me2s), in human glioblastoma and HEK293T cells. Consistently, both associated with protein Arginine Methyltransferase 5 (PRMT5)—the catalyst of the reaction—and its co-factor Methylosome Protein 50 (MEP50). Confocal experiments showed that Omomyc co-localized with c-Myc, PRMT5 and H4R3me2s-enriched chromatin domains. Finally, interfering with PRMT5 activity impaired target gene activation by Myc whereas it restrained Omomyc-dependent repression. The identification of a histone-modifying complex associated with Omomyc represents the first demonstration of an active role of this miniprotein in modifying chromatin structure and adds new information regarding its action on c-Myc targets. More importantly, the observation that c-Myc may recruit PRMT5-MEP50, inducing H4R3 symmetric di-methylation, suggests previously unpredictable roles for c-Myc in gene expression regulation and new potential targets for therapy.

Dysregulation of c-Myc—a basic helix-loop-helix/leucine zipper (bHLH-Zip) transcriptional regulator that controls a variety of normal cellular functions^1^—is a major mechanism of tumorigenesis. Abnormal levels of Myc (c-, N-, L-Myc) proteins are strongly associated with a variety of human cancers. c-Myc—hereafter named Myc—dimerizes with another member of the same family, Max; the heterodimer binds DNA with maximum selectivity for the E-box sequence CACGTG. Myc also interacts with a large number of proteins and multicomponent complexes involved in the regulation of transcription and chromatin structure. Unlike the majority of transcription factors, it does not stimulate transcription initiation; rather it enhances the production of transcripts from already active genes^2^,^3^,^4^, usually by promoting transcription elongation. It is still debated whether Myc directly represses a critical set of target genes or repression results from induction of factors like EZH2, a component of the Polycomb repressive complex^5^,^6^.

Myc represents an established target for cancer treatment, as demonstrated by studies in animal models, by drugs that mostly affect Myc transcription like JQ1^7^ and by Myc dominant negatives such as Omomyc, a ninety amino acid long, mutant bHLH-Zip domain^8^,^9^,^10^ that affects Myc function at the level of protein interactions and DNA binding^11^. Omomyc retains Myc transprepressive properties and displays high therapeutic efficacy in a variety of transgenic models—lung carcinoma^12^, SV40-driven pancreatic insulinoma^13^, glioma^14^—while being well tolerated for an extended period of time^12^. A further clarification of how Omomyc works at the cellular and mechanistic level in cancer cells is very relevant for developing strategies or designing small molecules able to interfere with Myc for cancer therapy.

Glioblastoma multiforme (GBM; WHO grade IV astrocytoma) is the most common and aggressive brain tumour in the adult, usually fatal in about 15 months^15^. GBM has the propensity to infiltrate, making complete surgical resection impossible, has a very heterogeneous cellular composition, and is largely resistant to radiation and chemotherapy^15^. This latter feature appears to depend on rare fractions of self-renewing, multi-potent cells able to proliferate and give origin to neuroepithelial lineages, named tumor initiating cells (TICs) or glioblastoma stem cells (GSCs)^16^. GSCs are capable of repopulating the tumour after treatment^16^ and are believed to be responsible for tumour progression and recurrence. Like other cancer stem cells, GSCs usually present Myc network activation^17^, which is required for GSC pool maintenance *in vitro* and tumorigenic potential *in vivo*^18^.

Dysregulation of epigenetic mechanisms is a distinct feature of cancer. In particular, both DNA and histone methylation profiles have been found to be altered in many tumours^19^,^20^. Histone methylation may occur at lysine (K) or arginine (R) residues. Histone R methylation has been less investigated than histone K methylation so far. However, growing evidences indicate protein arginine methyltransferases (PRMTs) as misregulated in cancer. PRMTs belong to the largest class (class I) of S-adenosylmethionine (AdoMet)-dependent methyltransferase enzymes and are further divided into 4 types, according to their methylation pattern (Wolff, 2009)^21^. PRMT5 is able to mono-methylate or symmetrically di-methylate non-histone and histone proteins^20^,^21^,^22^, being the latter usually repressive of transcription. PRMT5 expression and activity were found to be dysregulated in many tumors^20^; more recent data indicate PRMT5 as an important player in glioblastoma pathogenesis. Indeed, PRMT5 expression correlates with glioma malignant grade and is inversely related to patient survival^23^,^24^. Consistently, PRMT5 knock out decreases the proliferation rate of glioblastoma cells as well as their survival and migratory capacity^24^.

In the present work we show that Myc and Omomyc induce a pronounced, genome-wide, increase in PRMT5-dependent symmetric di-methylation of R3 on histone H4 (H4R3me2s), in 293T and glioblastoma cells. Consistently, Myc and Omomyc associated with the PRMT5-Methylosome 50 (MEP50)^25^ complex. Co-localization experiments confirmed the spatial overlapping of Omomyc, PRMT5, and Myc with H4R3me2s enriched chromatin regions. Further, inhibiting PRMT5 activity in the nucleus rescued Omomyc-dependent transcriptional repression of Myc target genes and impaired their activation by Myc.

Altogether, these data underpin a novel, unpredictable, PRMT5-based regulatory mechanism underlying Myc-dependent transcription. Further, they provide new information about Omomyc function at the molecular and epigenetic levels.

## Results

### Myc and Omomyc induce symmetric di-methylation of arginine 3 (R3) of histone H4

**Figure 1:**
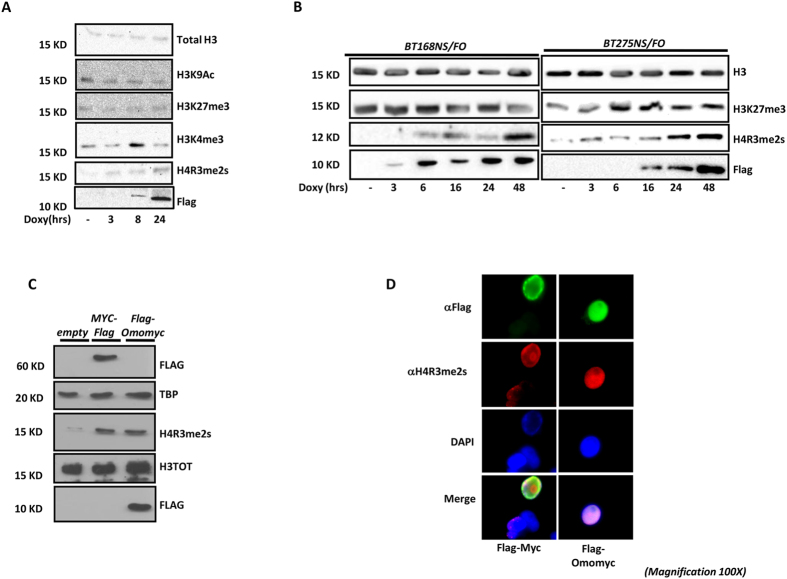
Omomyc and Myc-dependent H4R3 symmetric di-methylation. (**A**) Western blot analysis of U87MG/FO cell extracts along a time course between 3 and 24 hrs after doxycycline treatment. Picture shows the global changes of several histone modifications upon Omomyc expression. H3K9 histone acetylation decreased, as expected. H3K27 tri-methylation slightly decreased, as well. On the contrary, a peak of K4 tri-methylation on histone H3 was observed after 8 hrs of doxycycline treatment. The increase in H4R3 symmetric di-methylation followed the kinetic of expression of Flag-Omomyc. (**B**) Western blot analysis of BT168/FO and BT275/FO cell extracts along a time course between 3 and 48 hrs after doxycycline treatment. H4R3me2s increased in parallel with Flag-Omomyc. (**C**) Western blot analysis of nuclear extracts from HEK293T cells transfected with Flag-Omomyc and Flag-Myc encoding vectors. H4R3me2s was induced upon both Omomyc and Myc expression. The empty vector was used as control. (**D**) Double immunofluorescence. HEK293T cells transfected with either Flag-Omomyc or Flag-Myc enconding vectors were stained simultaneously with anti-H4R3me2s and anti-Flag antibodies. HEK293T cells expressing either Flag-Omomyc or Flag-Myc were also positive for H4R3me2s. These results are representative of at least three independent experiments.

Omomyc expression results in a marked deacetylation of lysine 9 (K9) on histone H3 (H3K9Ac) in Rat1 cells^11^. To further investigate the epigenetic effects of Myc inhibition by Omomyc in glioblastoma cells, we analysed the expression of several histone modifications upon Omomyc induction by doxycycline in U87MG cells infected with a lentivirus encoding a Flag-Omomyc (FO; U87MG/FO) fusion protein. We found that H3K9 histone acetylation decreased as expected. Among the other modifications tested, we observed an increase in H4R3me2s, which paralleled Omomyc expression (Fig. 1A). The same result was obtained in patient-derived glioblastoma stem cells BT168/FO and BT275/FO (Fig. 1B,C). To further validate these data and evaluate whether this epigenetic function was shared by Myc and Omomyc, we ectopically expressed Flag-Omomyc and Flag-Myc proteins in HEK293T cells, and analysed H4R3me2s by Western blot. We found that Flag-Omomyc and Flag-Myc induced H4R3me2s (Fig. 1C). This result attributes a specific role to Myc in regulating H4R3 symmetric di-methylation. Moreover, by immunofluorescence, we found that Flag-Omomyc and Flag-Myc expressing HEK293T cells were highly positive for H4R3me2s (Fig. 1D), compared to controls. Altogether, these results ascribe a specific role to Myc in regulating H4R3 symmetric di-methylation, a role that is maintained by Omomyc.

### Myc and Omomyc interact with PRMT5

**Figure 2:**
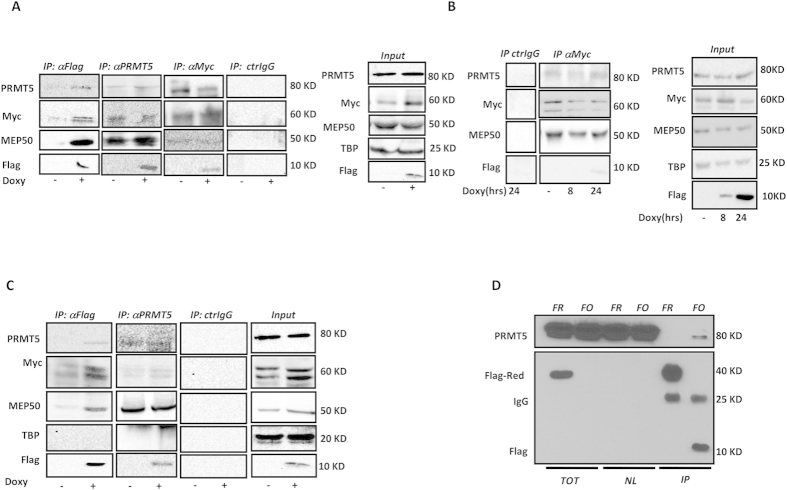
Omomyc and Myc associate to the PRMT5-MEP50 complex. (**A**) Immunoprecipitation experiments. Cell extracts from U87MG/FO cells—cultured in the presence or absence of doxycycline for 24 hrs—were immunoprecipitated with either anti-Flag, anti-PRMT5 or anti-Myc antibodies. Flag-Omomyc, PRMT5, MEP50 and Myc were detected by subsequent western blot analyses. Flag-Omomyc recruited PRMT5-MEP50 and Myc proteins. Consistently, PRMT5-MEP50 recruited both Flag-Omomyc and Myc. Immunoprecipitation with the anti-Myc antibody was less efficient; however, Flag-Omomyc and PRMT5-MEP50 were still detected. (**B**) Immunoprecipitation from BT168/FO cell extracts after 8 and 24 hrs of doxycycicline treatment. The anti-Myc antibody was used. PRMT5-MEP50, Flag-Omomyc and Myc were found associated also in this cell line. (**C**) Immunoprecipitation experiments from HEK293T/FO cell extracts. Either Anti-Flag or anti-PRMT5 antibodies were used. In both the experiments, Flag-Omomyc, PRMT5-MEP50 and Myc were found associated. (**D**) Immunoprecipitation experiments from HEK293T cell extracts transiently co-tranfected with both PRMT5-6myc and Flag-Omomyc (FO) vectors. The Flag-Red (FR) vector was used as control. PRMT5 was found specifically associated to Flag-Omomyc. Abbreviations: TOT = total extracts; NL = nuclear extracts; IP = immunoprecipitate. All these results are representative of at least four independent experiments.

Symmetric di-methylation of R3 on H4 is PRMT5-dependent. Therefore, we performed immunoprecipitation experiments, both in glioblastoma and HEK293T cells, to assess PRMT5 recruitment by Myc and Omomyc. A series of reciprocal immunoprecipitations, shown in Fig. 2A, revealed the formation of the same complex when U87MG/FO protein extracts were immuno precipitated with either anti-Flag, anti-PRMT5 or anti-Myc antibodies. BT168 cells present a high level of Myc (not shown). Therefore, immunoprecipitation experiments were also performed in this cell line by using the Myc antibody. As shown in Fig. 2B, Myc, Omomyc, and PRMT5 were found to be associated in BT168/FO cells as well. Similar results were obtained in HEK293T/FO cells (Fig. 2C). To further confirm these data, HEK293T cells were transiently co-transfected with 6myc-tagged PRMT5 and Flag-Omomyc expression vectors and immunoprecipitation were performed 48 hrs after transfection with an anti-Flag antibody. As shown in Fig. 2D, a sharp interaction between Omomyc and PRMT5 was found in HEK293T cells transfected with both plasmids.

### Omomyc co-localizes with Myc, PRMT5 and H4R3me2s-enriched chromatin domains in U87MG cells

**Figure 3:**
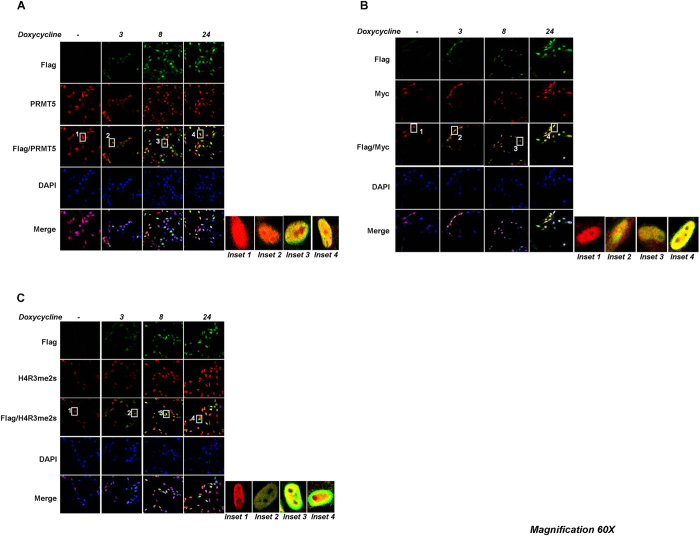
Omomyc co-localization with PRMT5, Myc and H4R3me2s-enriched chromatin regions in U87MG/FO cells. Confocal analyses showing an almost complete overlay between Flag-Omomyc, PRMT5 (**A**), c-Myc (**B**) and H4R3me2s (**C**) in U87MG/FO cells. Images are representative of at least 10 fields analyzed.

Co-localization between Omomyc, Myc, PRMT5 and H4R3me2s-enriched chromatin regions was evaluated by confocal analyses in U87MG/FO cells upon doxycycline treatment. Figure 3 shows an almost complete overlap between Omomyc—revealed by Flag antibody—and either PRMT5, H4R3me2s or Myc. Intriguingly, panel A shows that PRMT5 presented a diffuse cellular localization in untreated cells (see inset 1). Conversely, as long as Omomyc was expressed, PRMT5 clearly was placed in the nucleus (inset 2). Western blot analyses on fractionated cells extracts confirmed this observation (Supplementary Fig. S1). Myc co-localized with Omomyc in the nucleus, as expected (Fig. 3B). Importantly, Fig. 3C and supplementary Fig. S2 show that symmetric di-methylation of R3 on histone H4 paralleled Omomyc expression in both U87MG/FO and BT168/FO cells. In U87MG and BT168 wild type cells, cultured in the presence of doxycycline, no changes in symmetric di-methylation of R3 on histone H4 were found (Supplementary Figures S2 and S3). These data further validate Omomyc association with PRMT5 and confirm the ability of Omomyc to induce H4R3 symmetric di-methylation, presumably by recruiting a PRMT5-MEP50-containing complex.

### Inhibition of PRMT5 nuclear activity impairs target gene activation by Myc and repression by Omomyc

**Figure 4:**
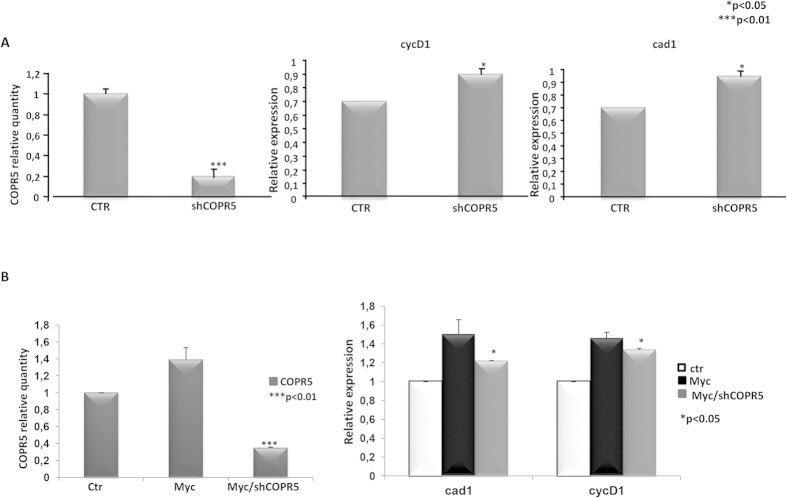
Interfering with PRMT5 nuclear activity restrained Omomyc-dependent repression and impairs activation of Myc targets (**A**) HEK293T/FO cells. CycD1 and cad gene expression was repressed for a 30% upon Omomyc induction. Their expression was almost completely recovered by Inhibiting PRMT5 through the ectopic expression of shCOPR5. (**B**) HEK293T wild type cells. Myc ectopic expression increased CycD1 and cad mRNA levels. The presence of shCOPR5, but not of a scrambled shRNA vector, weakened Myc-dependent cycD1 and cad activation.

PRMT5 catalytic activity in the nucleus requires at least two co-factors, the methylosome protein 50 (MEP50)^25^, associated with PRMT5 both in the cytoplasm and nucleus, and the cooperator of PRMT5 (COPR5)^26^, which selectively sustains PRMT5 nuclear function. Since PRMT5 shRNAs were ineffective to knock down PRMT5 transcripts, we used shRNAs against COPR5 to transfect HEK293T/FO cells, harbouring an inducible Omomyc. Real time PCR experiments were performed to evaluate the expression level of two target genes activated by Myc: carbamoyl-phosphate synthase aspartatecarbamoyltransferase-dihydroorotase^27^ (cad) and cyclin D1 (cycD1)^28^. Upon Omomyc induction, we observed a 30% inhibition of cad and cycD1 expression, which was recovered by co-transfection of shCOPR5 (Fig. 4A). This suggests that PRMT5 might be one of the molecules involved in Omomyc-dependent repression of these Myc activated genes. Moreover, ChIP experiments indicated a local enrichment of H4R3me2s histone residues at Omomyc-bound upstream chromatin domains of cycD1 and nucleolin (Ncl) gene promoters (Supplementary Fig. S4). We asked whether PRMT5 inhibition by shCOPR5 might affect Myc activation of the two targets and transfected HEK293T cells with plasmids expressing Myc and shCOPR5. We found that Myc transactivated cad and cycD1, as expected. Cotransfection of shCOPR5, instead, impaired transactivation by Myc (Fig. 4B).

These data strongly suggest that PRMT5 methylase complex and H4R3 symmetric di-methylation may modulate Myc activity at the epigenetic level, possibly by facilitating transactivation. Recruitment of the same methylase complex by Omomyc appeared, instead, associated with Omomyc-dependent transrepression of Myc targets, possibly by supporting deacetylation (Ref. 11 and Fig. 1A) and chromatin compaction.

## Discussion

PRMT5 plays a key role in a wide variety of cellular processes, including developmental pathways—such as neurogenesis^29^ and myogenesis^30^—metabolic responses^31^, somatic cell reprogramming^32^, and genome defense at the preimplantation stage^33^. In the cytoplasm, PRMT5 is involved in multiple functions, such as the regulation of Golgi apparatus^34^, ribosome biogenesis^35^, maintenance of pluripotency in embryonic stem cells^36^, neuronal spreading and migration^37^. PRMT5 acts in concert with a number of proteins involved in the epigenetic regulation of chromatin structure (for a comprehensive review see Karkhanis *et al.*, 2011)^38^. Several transcription factors interact with PRMT5^27^,^39^,^40^. Further, PRMT5 associates with N-Myc in neuroblastoma cells, promoting its stability, presumably by methylating a specific arginine (R242)^41^. However, no effect of the N-Myc/PRMT5 complex, either at epigenetic or at the transcription level, was reported. In the present study, we show that, in GBM cells, either U87MG or GSCs, as well as in HEK293T cells, Myc and Omomyc induce symmetrical H4R3 di-methylation (Fig. 1A–C). This effect correlates with the local enrichment of H4R3me2s histones and Omomyc at the chromatin domains upstream of Myc target gene promoters (Supplementary Fig. S4). In this view, Omomyc appears to simply retain a function of Myc. However, high levels of Myc and PRMT5 correlate with malignancy^23^,^38^, while Omomyc harbours anti-cancer properties. Thus, it is conceivable that, although associating both with PRMT5, the functional effect may be different. Indeed, inhibition of PRMT5 activity by a shRNA against its co-factor COPR5 restrained Myc transactivating properties, while recovered Omomyc-dependent repression of Myc targets (Fig. 4). PRMT5 interacts with a number of epigenetic enzymes involved in either activation or inhibition of transcription. It was recently associated to the activation of transcription of adipogenic genes, through H3R8 symmetric di-methylation and SWI/SNF chromatin-remodeling enzyme recruitment^42^. PRMT5-mediated symmetric di-methylation of H3R2 keeps genes poised for transcriptional activation^22^. Further, PRMT5 interacts with mSin3A/histone deacetylase (HDAC) complex^30^ and its activity mediates DNA methyltransferase 3a (Dnmt3a) binding and subsequent DNA methylation of H4R3me2s rich chromatin regions^43^. Restraining PRMT5 nuclear activity led to a recovery of Myc inhibition by Omomyc, suggesting that Omomyc may silence—at least in part—Myc target genes^11^ through PRMT5 recruitment and H4R3 symmetric di-methylation. At genomic level, Omomyc occupies Myc binding sites (Galardi *et al.*, submitted). Moreover, Omomyc globally induces histone deacetylation (Fig. 1A) while Myc promotes acetylation^44^. These findings suggest that complexes associated with either Myc or Omomyc on chromatin, although involving PRMT5 in both cases, may have a different activity. Further experiments are required to clarify these aspects. Indeed, both PRMT5 and Myc expression correlate with tumour malignancy^23^,^38^,^3^ and PRMT5 stabilizes N-Myc^41^ in neuroblastoma. We consistently found an association between Myc or Omomyc and PRMT5 in glioblastoma cells (Fig. 2). Based on this finding, we propose that Omomyc may perturb the Myc/PRMT5 interaction and that this may have a role in its potent anti-oncogenic properties.

A PRMT1/PRMT5/MEP50 containing complex was found to be associated with the Chromatin Target of PRMT1 (CHTOP) protein in glioblastoma cells and to play a critical role in the activation of cancer-related genes^45^. Thus, it is possible that Omomyc may affect the PRMT1/PRMT5/MEP50/CHTOP complex, impairing cancer-related gene expression.

PRMT5 is present both in the cytoplasm and in the nucleus of cancer cells. Its cytosolic localization has been associated with a high grade of malignancy. Indeed, cytoplasmic PRMT5 correlates with a poor prognosis in prostate^46^, melanoma^47^ and lung cancers^48^. In GBM cells, PRMT5 showed a preferential nuclear localization, although a sizeable fraction was distributed in the cytoplasm as well; interestingly, PRMT5 apparently localizes exclusively in the nucleus of normal neuronal cells^23^,^29^. Upon Omomyc induction, PRMT5 was present almost exclusively in the nucleus, where it co-localized with Myc, Omomyc, and H4R3me2s-enriched chromatin regions (Fig. 3). This data was further confirmed by western blot on fractionated cell extracts. In this context, Omomyc may represent a nuclear shuttle for PRMT5, eliminating the residual cytoplasmic amount, which may contribute to GBM tumorigenesis.

Altogether, these data report a newly identified functional interaction between Myc and PRMT5, adding to Myc a new layer of regulation at the epigenetic level. Further investigations are required to understand the biological significance of this association, which may envisage small molecule-based epigenetic therapies for GBM. Further, they suggest a PRMT5-based molecular mechanisms supporting Omomyc function as a transcriptional repressor of Myc target genes in GBM.

## Material and Methods

### Cell culture and treatment

Patient-derived brain tumor 168 (BT168) and brain tumor 275 stem cells (BT275) were cultured as described^49^. U87MG (ATCC-HTB14) and HEK293T cells were cultured in Dulbecco’s Modified Eagle Medium (DMEM, SIGMA, St. Louis, Mo, USA), supplemented with 10% Fetal Bovine Serum (FBS, Thermo Fisher Scientific, Waltham, MA, USA), 2 mM Glutammine and penicillin/streptomycin/amphotericin B (Thermo Fisher Scientific). To induce Omomyc expression, 0.25 μg/ml of doxycycline (SIGMA) were used.

### Lentiviral infection

U87MG/FlagOmomyc (FO), BT168/FO, BT275/FO and HEK293T/FO cells were all obtained by lentiviral transduction. Viral particles were produced in HEK293T cells by transient transfection using Lipofectamine 2000 reagent (Thermo Fisher Scientific). 2 × 10^6^ cells were cotransfected with 4 μg of pSLIK-FlagOmomyc, 2 μg each of the second-generation packaging plasmids PLP1 and PLP2 and 1 μg of the vesicular stomatitis virus (VSV) G envelope plasmid pMD VSV-G diluted in Opti-MEM (Thermo Fisher Scientific). The medium was removed after 12–16 h and replaced with 4 ml of fresh growth media. Supernatants were collected every 24 hr between 48 to 72 hr after transfection, pulled together and concentrated by ultracentrifugation in a Beckman SW-28 rotor for 2 hours at 25000 rpm, 4 °C. For lentiviral infection, 2–5 × 10^5^ cells were seeded in 35 mm dishes and infected the following day in cell culture medium containing 4 μg/ml polybrene. To generate stable inducible lines, cells were selected for two weeks with 50–200 μg/ml Hygromycin B (SIGMA). After selection, induction of FLAG-Omomyc expression by 0.25 μg/ml doxycycline (SIGMA) was assessed by both immunocytochemistry and western blotting.

### Plasmids and transfections

The Flag-Omomyc insert was excised from pCbsFlag-Omomyc plasmid with BamHI and HindIII restriction enzymes and inserted into pLNCX2 (Clontech, Saint-Germain-en-Laye France) using BglII-HindIII restriction sites. Flag-Myc was excised from the pCbsFlag-Myc vector and inserted in pLPCX (Clontech) in BamHI-ClaI restriction sites. The PRTM5 coding sequence was obtained from a human cDNA library by PCR amplification, and inserted in pCS2 vector^50^. pRFP-C-RS plasmid expressing a COPR5 specific shRNA sequence (shCOPR5) and the relative scrambled control were purchased by OriGene (Origene, Rockville, MD, U.S.A). Transfections were performed by using Lipofectamine 2000 (Thermo Fisher Scientific) according to the manufacturer’s instructions.

### Acid extraction of histones

Cells were lysed in 10 mM Hepes pH 7.9, 10 mM KCl, 0.2 mM EDTA, 1 mM DTT, 0.6% Nonidet-P40 and protease/phosphatase inhibitors cocktails (SIGMA). Lysates were incubated 15 minutes on ice and centrifuged at 10000 rpm for 10 minutes at 4 °C. The cytoplasmic fraction was recovered and stored at −80 °C. The nuclear pellet was resuspended in 20 mM Hepes pH 7.9, 0.4 M NaCl, 2 mM EDTA, 1 mM DTT and protease/phosphatase inhibitors cocktails. Samples were incubated on ice for 30 minutes and centrifuged at 13000 rpm for 10 minutes. The supernatant containing nuclear proteins was stored at –80 °C. The remaining pellet, containing DNA and proteins tightly associated, was washed in 10 mM NaCl, 10 mM Tris-HCl pH 7.5, 1.5 mM MgCl_2_ and finally resuspended in the same washing buffer added with 0.4N HCl to obtain HCl soluble proteins. Samples were incubated for at least 1 hour at 4 °C and the day after were centrifuged at 10000 rpm for 20 minutes at 4 °C. Supernatant was recovered and the acid-soluble proteins were precipitated with 10 volumes of acetone, over night (O/N) at −20 °C. The day after, samples were centrifuged at 10000 rpm for 20 minutes at 4 °C. Precipitated HCl-soluble proteins were resuspended in sterile H_2_O and stored at −20 °C.

### Fractionated cell extracts

Fractionated cell extracts were performed as described^51^.

### Immunoprecipitation

For immunoprecipitation experiments, cells were lysed in 10 mM Hepes pH 7.9, 50 mM NaCl, 500 mM Sucrose, 0.1 mM EDTA, 0.5% Nonidet-P40, 5 mM MgCl_2_, 1 mM DTT and protease/phosphatase inhibitors cocktails. Samples were incubated on ice for 30 minutes and centrifuged at 8000 rpm at 4 °C. The cytoplasmic faction was recovered and stored on ice. Nuclei were resuspended in 20 mM Hepes pH 7.9, 0.42 M NaCl, 25% Glycerol, 0.2 mM EDTA, 1.5 mM MgCl_2_, 0.5 mM DTT and protease/phosphatase inhibitors cocktails. Samples were vortexed for 10 seconds and centrifuged for 5 minutes at 14000 rpm at 4 °C. Supernatants containing nuclear proteins were recovered, mixed with the previously collected cytoplasmic fraction and the mixture underwent immunoprecipitation. At least 1 mg of extract was used for each experiment. Immunoprecipitation was performed by using either ImmunoCruzF or C system (Santa Cruz Biotechnologies, Santa Cruz, CA, USA), according to the manufacturer’s instructions.

### Western blot

Proteins were resolved in 12% polyacrilammide gels and blotted onto nitrocellulose filters (GE Heath Care, Little Chafont, Buckinghamshire, UK) for 2 hours at 250 mA on ice. Filters were blocked in phosphate buffered saline plus 0.1% Tween-20 (PBST, SIGMA) added with 10% non-fat dry milk, for 1 hour at room temperature (RT). Primary antibodies were incubated O/N at 4 °C, according to the concentration recommended by the manufacturer, in PBST plus 2.5% non-fat dry milk. After three 5 minutes washes, filters were incubated for 1 hour at RT with either goat-anti rabbit (1:5000) or goat-anti mouse (1:2000) horseradish peroxidase (HRP)-conjugated secondary antibodies (Merck Millipore, Darmstadt, Germany), or with ImmunoCruz F or C detection reagent, in the case of immunoprecipitated proteins, in PBST plus 2.5% non-fat dry milk. Proteins were revealed by SuperSignal West Pico Chemiluminescent Substrate (Thermo Fisher Scientific). Images were captured with a Chemidoc XRS^+^ (Bio-Rad, Hercules, CA, USA).

Anti-H4R3me2s, anti-MEP50 and anti-PRMT5 antibodies were from Abcam (Abcam, Cambridge, UK); anti-Flag and anti-H3 antibodies were from SIGMA. Anti-H3K4me3, anti-H3K27me3 and anti-H3K9Ac antibodies were from Merck-Millipore. Anti-Myc and anti-TATA Binding Protein (TBP) were from Santa Cruz Biotechnologies.

### Immunofluorescence

Immunofluorescence analysis on U87MG cells was performed as described^11^. Immunofluorescence on BT168 cells, grown as neurospheres, was performed as follows. Neurospeheres were centrifuged for 20 minutes at 400 rpm, washed in PBS 1X and centrifuged again for 5 minutes at 800 rpm. Cells were transferred to a microcentrifuge tube and fixed in 4% paraformaldheyde for 15 minutes at RT. Thereafter, neurospheres were centrifuged for 5 minutes at 800 rpm and autofluorescence was quenched with 25 mM Glycine in PBS 1X, pH 7.4, for 5 minutes. After two washes in PBS 1X, neurospheres were permeabilized with 0.3% Triton X-100 in PBS 1X for 5 minutes and washed with PBS/0.1% Tween-20. Blocking was in 10% BSA in PBS 1X, for either 1 hr at RT or O.N. at +4 °C. After a 5 minutes centrifugation at 800 rpm, primary antibodies were added in blocking solution O.N. Neurospheres were washed twice in PBS/0.1%Tween-20. Secondary antibodies were added in blocking solution for 1 hr. After two washes in PBS/0.1%Tween-20, neurospheres were counterstained with DAPI (1 μg/ml) in PBS 1X for 5 minutes and washed again in PBS/0.1% Tween-20. Supernatant was carefully removed and Neurospheres were resuspended in phosphate-buffered glycerol. Neurospheres were spotted onto microscope slides by using 20 μl pipette tips, cut to avoid neurospheres disruption. Secondary antibodies were: donkey-anti mouse AlexaFluor488 and goat-anti rabbit AlexaFluor555 (Thermo Fisher Scientific).

### Confocal analysis

Confocal analysis was performed as described^51^. Secondary antibodies were: donkey-anti mouse AlexaFluor488 and goat-anti rabbit AlexaFluor555 (Thermo Fisher Scientific).

### shRNA and real time PCR

shCOPR5 and scrambled control vector were transfected in HEK293T/FO by using Lipofectamine 2000 reagent (Thermo Fisher Scientific) according to the manufacturer’s instructions. Where indicated, HEK293T cells were treated for additional 16 hours with doxycycline. Total RNA was extracted by using TRIZOL reagent (Thermo Fisher Scientific) according to the manufacturer’s instructions. Reverse transcription reaction was performed by using M-MLV Reverse Transcriptase kit (Promega, Madison, WI, U.S.A). Expression levels of Omomyc target genes were analysed using SYBR Green- based Real Time RT-PCR (Thermo Fisher Scientific). Primers were:

hTBP F 5′ tgc ccg aaa cgc cga ata taa tc 3′

hTBP R 5′ tgg ttc gtg gct ctc tta tcc tc 3′

hCOPR5 F 5′gga gct gtc aga agg gac aa 3′

hCOPR5 R 5′ atg ctc tcc tgg atg tcg tc 3′

hcycD1 F 5′ gaa gat cgt cgc cac ctg 3′

hcycD1 R 5′ gac ctc ctc ctc gca ctt ct 3′

hCAD1 F 5′ cac tga gca tgg cgt caa 3′

hCAD1 R 5′ agc tgc tcc agg atg ctc 3′.

### Chromation Immunoprecipitation

Chromatin immunoprecipitation was performed as described^51^,^52^. Oligonucleotides were:

hCCND1prom-4129F GCTTTCCATTCAGAGGTGTGTTT (-871 from TSS)

hCCND1prom-4228R CTACCTTGACCAGTCGGTCCTT (-722 bp from TSS)

hNCLprom-4390F TCACAGAAAACCTCGCACAGA (-610 bp from TSS)

hNCLprom-4524R CCGTTGGCCCTTTTGGA (-476 bp from TSS).

## Additional Information

**How to cite this article**: Mongiardi, M. P. *et al.* Myc and Omomyc functionally associate with the Protein Arginine Methyltransferase 5 (PRMT5) in glioblastoma cells. *Sci. Rep.* **5**, 15494; doi: 10.1038/srep15494 (2015).

## Supplementary Material

Supplementary Information

## References

[b1] Eilers M. & Eisenman R. N. Myc’s broad reach. Genes Dev. 22, 2755–2766 (2008). 18923074 10.1101/gad.1712408 PMC2751281

[b2] Rahl P. B. *et al.* Myc regulates transcriptional pause release. Cell. 141, 432–445 (2010). 20434984 10.1016/j.cell.2010.03.030 PMC2864022

[b3] Nie Z. *et al.* c-Myc is a universal amplifier of expressed genes in lymphocytes and embryonic stem cells. Cell. 151, 68–79 (2012). 23021216 10.1016/j.cell.2012.08.033 PMC3471363

[b4] Lin C. Y. *et al.* Transcriptional amplification in tumor cells with elevated c-Myc. Cell. 151, 56–67 (2012). 23021215 10.1016/j.cell.2012.08.026 PMC3462372

[b5] Kaur M. & Cole M. D. MYC acts via the PTEN tumor suppressor to elicit autoregulation and genome-wide gene repression by activation of the Ezh2 methyltransferase. Cancer Res. 73, 695–705 (2013). 23135913 10.1158/0008-5472.CAN-12-2522 PMC3549058

[b6] Kuser-Abali G., Alptekin A. & Cinar B. Overexpression of MYC and EZH2 cooperates to epigenetically silence MST1 expression. Epigenetics. 9, 634–643 (2014). 24499724 10.4161/epi.27957 PMC4121373

[b7] Bandopadhayay P. *et al.* BET bromodomain inhibition of MYC-amplified medulloblastoma. Clin Cancer Res. 20, 912–925 (2014). 24297863 10.1158/1078-0432.CCR-13-2281 PMC4198154

[b8] Soucek L. *et al.* Inhibition of Myc family proteins eradicates KRas-driven lung cancer in mice. Genes Dev. 27, 504–513 (2013). 23475959 10.1101/gad.205542.112 PMC3605464

[b9] Soucek L. *et al.* Design and properties of a Myc derivative that efficiently homodimerizes. Oncogene. 17, 2463–2472 (1998). 9824157 10.1038/sj.onc.1202199

[b10] Soucek L. *et al.* Omomyc, a potential Myc dominant negative, enhances Myc-induced apoptosis. Cancer Res. 62, 3507–3510 (2002). 12067996

[b11] Savino M. *et al.* The action mechanism of the Myc inhibitor termed Omomyc may give clues on how to target Myc for cancer therapy. PLoS One. 6, e22284 (2011). 21811581 10.1371/journal.pone.0022284 PMC3141027

[b12] Soucek L. *et al.* Modelling Myc inhibition as a cancer therapy. Nature. 455, 679–683 (2008). 18716624 10.1038/nature07260 PMC4485609

[b13] Sodir N. M. *et al.* Endogenous Myc maintains the tumor microenvironment. Genes Dev. 25, 907–916 (2011). 21478273 10.1101/gad.2038411 PMC3084025

[b14] Annibali D. *et al.* Myc inhibition is effective against glioma and reveals a role for Myc in proficient mitosis. Nat Commun. 5, 4632 (2014). 25130259 10.1038/ncomms5632 PMC4143920

[b15] Preusser M. *et al.* Current Concepts and Management of Glioblastoma. Ann Neurol. 70, 9–21 (2011). 21786296 10.1002/ana.22425

[b16] Piccirillo S. G. & Vescovi A. L. Brain tumour stem cells: possibilities of new therapeutic strategies. Expert Opin Biol Ther. 7, 1129–35 (2007). 17696813 10.1517/14712598.7.8.1129

[b17] Kim J. *et al.* Myc network accounts for similarities between embryonic stem and cancer cell transcription programs. Cell. 143, 313–324 (2010). 20946988 10.1016/j.cell.2010.09.010 PMC3018841

[b18] Wang J. *et al.* c-Myc is required for maintenance of glioma cancer stem cells. PloS One. 3, e3769 (2008). 19020659 10.1371/journal.pone.0003769 PMC2582454

[b19] Feinberg A. P. & Vogelstein B. Hypomethylation distinguishes genes of some human cancers from their normal counterparts. Nature. 301, 89–92 (1983). 6185846 10.1038/301089a0

[b20] Yost J. M., Korboukh I., Liu F., Gao C. & Jin J. Targets in epigenetics, inhibiting the methyl writers of the histone code. Curr Chem Genomics. 5(Suppl 1), 72–84 (2011). 21966347 10.2174/1875397301005010072 PMC3178896

[b21] Wolf S. S. The protein arginine methyltransferase family, An update about function, new perspectives and the physiological role in humans. Cell Mol Life Sci. 66, 2109–2121 (2009). 19300908 10.1007/s00018-009-0010-x PMC11115746

[b22] Migliori V. *et al.* Symmetric dimethylation of H3R2 is a newly identified histone mark that supports euchromatin maintenance. Nat Struct Mol Biol. 19, 136–144 (2012). 22231400 10.1038/nsmb.2209

[b23] Han X. *et al.* Expression of PRMT5 correlates with malignant grade in gliomas and plays a pivotal role in tumor growth *in vitro*. J Neurooncol 118, 61–72 (2014). 24664369 10.1007/s11060-014-1419-0 PMC4076054

[b24] Yan F. *et al.* Genetic validation of the protein arginine methyltransferase PRMT5 as a candidate therapeutic target in glioblastoma. Cancer Re. 74, 1752–1765 (2014). 10.1158/0008-5472.CAN-13-0884 PMC3959215 24453002

[b25] Friesen W. J. *et al.* A novel WD repeat protein component of the methylosome binds Sm proteins. J Biol Chem. 277, 8243–8247 (2002). 11756452 10.1074/jbc.M109984200

[b26] Lacroix M. *et al.* The histone-binding protein COPR5 is required for nuclear functions of the protein arginine methyltransferase PRMT5. EMBO Rep. 9, 452–458 (2008). 18404153 10.1038/embor.2008.45 PMC2373370

[b27] Pal S. *et al.* mSin3A/histone deacetylase 2- and PRMT5-containing Brg1 complex is involved in transcriptional repression of the Myc target gene cad. Mol Cell Biol. 23, 7475–7487 (2003). 14559996 10.1128/MCB.23.21.7475-7487.2003 PMC207647

[b28] Daksis J. I., Lu R. Y., Facchini L. M., Marhin W. W. & Penn L. J. Myc induces cyclin D1 expression in the absence of *de novo* protein synthesis and links mitogen-stimulatedsignal transduction to the cell cycle. Oncogene. 9, 3635–3645 (1994). 7526316

[b29] Chittka A., Nitarska J., Grazini U. & Richardson W. D. Transcription factor positive regulatory domain 4 (PRDM4) recruits protein arginine methyltransferase 5 (PRMT5) to mediate histone arginine methylation and control neural stem cell proliferation and differentiation. J Biol Chem. 287, 42995–43006 (2012). 23048031 10.1074/jbc.M112.392746 PMC3522294

[b30] Dacwag C. S., Ohkawa Y., Pal S., Sif S. & Imbalzano A. N. The protein arginine methyltransferase Prmt5 is required for myogenesis because it facilitates ATP-dependent chromatin remodeling. Mol Cell Biol. 27, 384–394 (2007). 17043109 10.1128/MCB.01528-06 PMC1800640

[b31] Tsai W. W. *et al.* PRMT5 modulates the metabolic response to fasting signals. Proc Natl Acad Sci USA. 110, 8870–8875 (2013). 23671120 10.1073/pnas.1304602110 PMC3670393

[b32] Nagamatsu G. *et al.* A germ cell-specific gene, Prmt5, works in somatic cell reprogramming. J Biol Chem. 286, 10641–10648 (2011). 21270127 10.1074/jbc.M110.216390 PMC3060515

[b33] Kim S. *et al.* PRMT5 Protects Genomic Integrity during Global DNA Demethylation in Primordial Germ Cells and Preimplantation Embryos Mol Cell. 56, 564–579 (2014). 25457166 10.1016/j.molcel.2014.10.003 PMC4250265

[b34] Zhou Z. *et al.* PRMT5 regulates Golgi apparatus structure through methylation of the golgin GM130. Cell Res. 20, 1023–1033 (2010). 20421892 10.1038/cr.2010.56

[b35] Ren J. *et al.* Methylation of ribosomal protein S10 by protein arginine methyltransferase 5 regulates ribosome biogenesis. J Biol Chem. 285, 12695–12705 (2010). 20159986 10.1074/jbc.M110.103911 PMC2857073

[b36] Tee W. W. *et al.* Prmt5 is essential for early mouse development and acts in the cytoplasm to maintain ES cell pluripotency. Genes Dev. 24, 2772–2777 (2010). 21159818 10.1101/gad.606110 PMC3003195

[b37] Guo S. & Bao S. srGAP2 arginine methylation regulates cell migration and cell spreading through promoting dimerization. J Biol Chem. 285, 35133–3541 (2010). 20810653 10.1074/jbc.M110.153429 PMC2966127

[b38] Karkhanis V., Hu Y. J., Baiocchi R. A., Imbalzano A. N. & Sif S. Versatility of PRMT5-induced methylation in growth control and development. Trends Biochem Sci. 36, 633–641 (2011). 21975038 10.1016/j.tibs.2011.09.001 PMC3225484

[b39] Ancelin K. *et al.* Blimp1 associates with Prmt5 and directs histone arginine methylation in mouse germ cells. Nat Cell Biol. 8, 623–630 (2006). 16699504 10.1038/ncb1413

[b40] Sankaran V. G., Xu J. & Orkin S. H. Advances in the understanding of haemoglobin switching. Br J Haematol. 149, 181–194 (2010). 20201948 10.1111/j.1365-2141.2010.08105.x PMC4153468

[b41] Park J. H. *et al.* Protein arginine methyltransferase 5 is a key regulator of the MYCN oncoprotein in neuroblastoma cells. Mol Oncol. 9, 617–627 (2015). 25475372 10.1016/j.molonc.2014.10.015 PMC4359099

[b42] LeBlanc S. E. *et al.* Protein arginine methyltransferase 5 (Prmt5) promotes gene expression of peroxisome proliferator-activated receptor γ2 (PPARγ2) and its target genes during adipogenesis. Mol Endocrinol. 26, 583–597 (2012). 22361822 10.1210/me.2011-1162 PMC3327358

[b43] Zhao Q. *et al.* PRMT5-mediated methylation of histone H4R3 recruits DNMT3A, coupling histone and DNA methylation in gene silencing. Nat Struct Mol Biol 16, 304–311 (2009). 19234465 10.1038/nsmb.1568 PMC5120857

[b44] Ullius A. *et al.* The interaction of MYC with the trithorax protein ASH2L promotes gene transcription by regulating H3K27 modification. Nucleic Acids Res. 42, 6901–6920 (2014). 24782528 10.1093/nar/gku312 PMC4066752

[b45] Takai H. *et al.* 5 Hydroxymethylcytosine plays a critical role in glioblastomagenesis by recruiting the CHTOP-methylosome complex. Cell Rep. 9, 48–60 (2014). 25284789 10.1016/j.celrep.2014.08.071

[b46] Gu Z. *et al.* Protein arginine methyltransferase 5 functions in opposite ways in the cytoplasm and nucleus of prostate cancer cells. PLoS One 7, e44033 (2012). 22952863 10.1371/journal.pone.0044033 PMC3428323

[b47] Nicholas C. *et al.* PRMT5 is upregulated in malignant and metastatic melanoma and regulates expression of MITF and p27(Kip1). PLoS One. 8, e74710 (2013). 24098663 10.1371/journal.pone.0074710 PMC3786975

[b48] Ibrahim R. *et al.* Expression of PRMT5 in lung adenocarcinoma and its significance in epithelial-mesenchymal transition. Hum Pathol. 45, 1397–1405 (2014). 24775604 10.1016/j.humpath.2014.02.013

[b49] De Bacco F. *et al.* The MET oncogene is a functional marker of a glioblastoma stem cell subtype. Cancer Res. 72, 4537–4550 (2012). 22738909 10.1158/0008-5472.CAN-11-3490

[b50] Rupp R. A., Snider L. & Weintraub H. Xenopus embryos regulate the nuclear localization of XMyoD. Genes Dev. 8, 1311–1323 (1994). 7926732 10.1101/gad.8.11.1311

[b51] Illi B. *et al.* Nitric oxide modulates chromatin folding in human endothelial cells via protein phosphatase 2A activation and class II histone deacetylases nuclear shuttling Circ Res. 102, 51–58 (2008). 17975112 10.1161/CIRCRESAHA.107.157305

[b52] Nanni S. *et al.* Endothelial NOS, estrogen receptor beta, and HIFs cooperate in the activation of a prognostic transcriptional pattern in aggressive human prostate cancer. J Clin Invest. 119, 1093–10108 (2009). 19363294 10.1172/JCI35079 PMC2673846

