# Supplementary material for: Myc and Omomyc functionally associate with the Protein Arginine Methyltransferase 5 (PRMT5) in glioblastoma cells
Source: Sci Rep. 2015 Nov 13;5:15494. doi: 10.1038/srep15494 (PMC4643314; doi:10.1038/srep15494)
Supplement: Supplementary Information [file srep15494-s1.pdf]

Supplementary Information

Myc and Omomyc functionally associate with the Protein Arginine Methyltransferase 5 (PRMT5) in glioblastoma cells

Maria Patrizia Mongiardi, Mauro Savino, Laura Bartoli, Sara Beji, Simona Nanni, Fiorella Scagnoli, Maria Laura Falchetti, Annarita Favia, Antonella Farsetti, Andrea Levi, Sergio Nasi, Barbara Illi.

Supplementary Figure Legend

Supplementary Figures

Supplementary figure S1

Western blot on U87MG fractionated cell extracts. In U87MG/FO cells, PRMT5 nuclear translocation is detected upon doxycycline treatment. This result is representatrive of two independent experiments. N= nucleus; C= cytoplasm.

Supplementary figure S2

Immunofluorescence analyses. BT168 wild type (wt) and BT168/FO cells, cultured in the presence or absence of doxycycline for 24 hrs, were stained with anti-Flag/anti-H4R3me2s antibodies. Neither Flag staining nor appreciable differences in the intensity of H4R3me2s staining were detected in wt cells, as expected. Conversely, in BT168/FO cells, the level of H4R3me2s increased upon Omomyc induction.

Supplementary figure S3

A) Confocal analyses. U87MG wild type (wt) cells, cultured in the presence or absence of doxycycline for 24 hrs, were stained with anti-Flag/anti-PRMT5 (left), anti-Flag/anti-Myc (middle), anti-Flag/anti-H4R3me2s (right) antibodies. No Flag staining was observed, as expected. No appreciable differences in the expression and/or subcellular localization of PRMT5, Myc and H4R3me2s were detected. At the bottom, right, a representative western blot analysis on U87MG wt cell extracts, evaluating H4R3me2s levels is shown. No differences in H4R3me2s were observed upon doxycycline treatment.

Supplementary figure S4

Chromatin immunoprecipitation. Chromatin from U87MG/FO cells, cultured in the presence or absence of doxycycline for 24 hours, was immunoprecipitated with either anti-Flag, anti-H4R3me2s

or anti-H3K9me3 antibodies. Chromatin incubated with sepharose beads alone was used as control. Flag-Omomyc and H4R3me2s accumulated at human Nucleolin (hNCL) and CycD1 (hCycD1) promoters upon doxycycline treatment. No relevant differences were observed in H3K9me3 local enrichment, with or without doxycycline. Recruitment onto promoters was detected by qPCR (see Methods) and expressed as Relative Enrichment in arbitrary units (A.U.).

Fig. S1

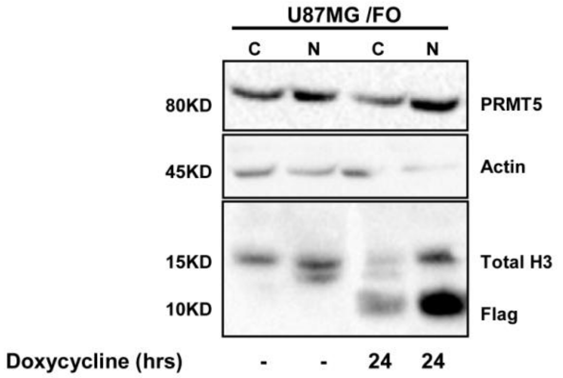
U87MG /FO
C N C N
80KD
PRMT5
45KD
Actin
15KD
Total H3
10KD
Flag
Doxycycline (hrs)
- - 24 24

Fig. S2

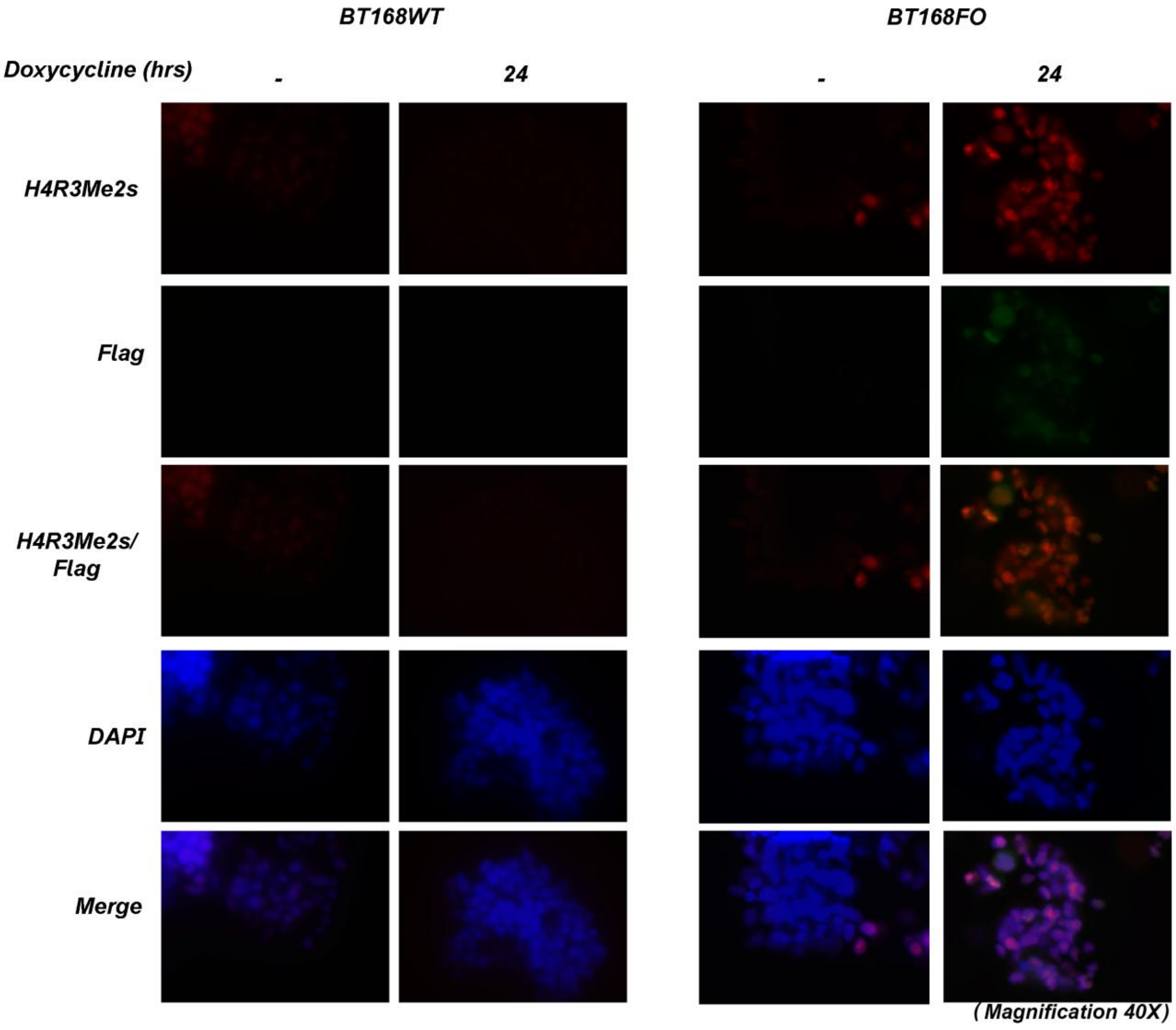
BT168WT
BT168FO
Doxycycline (hrs)
-
24
-
24
H4R3Me2s
Flag
H4R3Me2s/ Flag
DAPI
Merge
(Magnification 40X)

Fig. S3

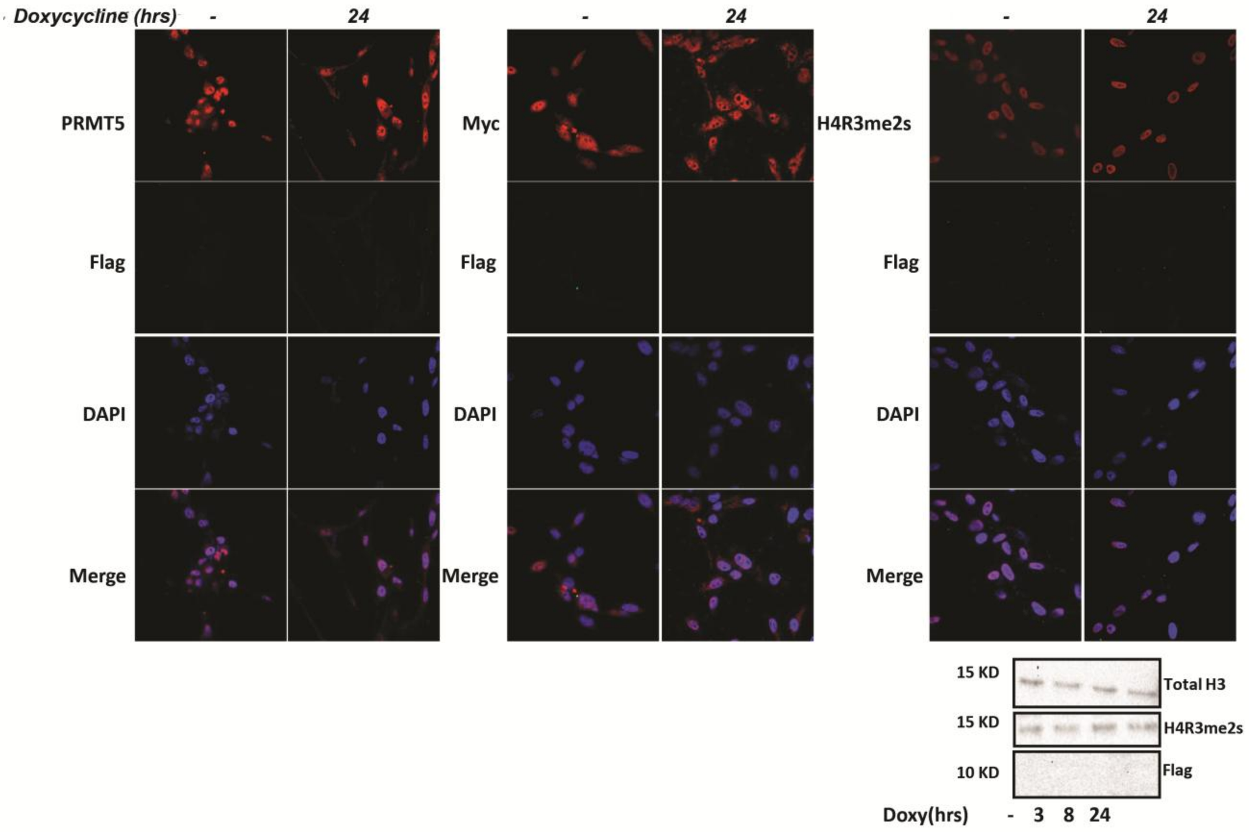

(Magnification 60X)

Fig. S4

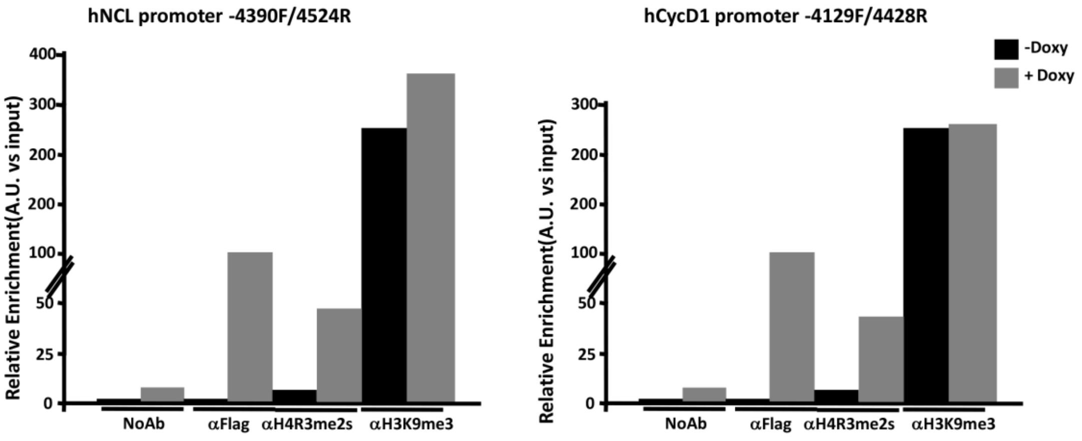
hNCL promoter -4390F/4524R
hCycD1 promoter -4129F/4428R
-Doxy
+ Doxy
Relative Enrichment(A.U. vs input)
400
300
200
200
100
50
25
0
300
200
200
100
50
25
0
NoAb
αFlag
αH4R3me2s
αH3K9me3
NoAb
αFlag
αH4R3me2s
αH3K9me3
